# The Mediating Role of Emotional Arousal and Donation Anxiety on Blood Donation Intentions: Expanding on the Theory of Planned Behavior

**DOI:** 10.3390/bs14030242

**Published:** 2024-03-17

**Authors:** Stefanos Balaskas, Maria Koutroumani, Maria Rigou

**Affiliations:** Department of Management Science and Technology, School of Economics and Business, University of Patras, 26334 Patras, Greece; mary.koutroumani@gmail.com (M.K.); rigou@upatras.gr (M.R.)

**Keywords:** blood donation intentions, theory of planned behavior (TPB), blood donation anxiety, emotional arousal, structural equation modelling

## Abstract

Blood donation is essential in health-care systems worldwide, dealing with the demand for transfusions, and for the treatment of a variety of medical conditions. A major obstacle is raising the rate of blood donations by recruiting and retaining donors in an efficient manner. This paper presents a comprehensive analysis of blood donation, utilizing an enhanced framework based on the theory of planned behavior with an emphasis on emotional arousal (positive and negative), attitudes towards advertisements, and blood donation anxiety, revealing critical psychological and communicative determinants of blood donation intention. To achieve this, a quantitative non-experimental correlational technique was employed to collect data from 414 individuals using an online questionnaire circulated across Greek society. The data were analyzed using structural equation modelling, with a focus on the direct impacts on donation intentions and the role of emotional arousal as a mediator. The findings indicate that attitudes and anxiety have strong direct impacts on the behavioral intention to donate, underlining the important barriers generated by donation anxieties as well as the efficacy of positive attitudes and successful advertising. Furthermore, the study demonstrates emotional arousal as a partial mediator, implying that both cognitive assessments and emotional responses play a role in influencing donation intentions. This study takes on a new approach to give emphasis and provide evidence of the mediating effect of emotional arousal on donation intention, utilizing structural equation modeling. Despite the critical role of marketing as a primary source of blood donors, the implementation of emotional marketing techniques has been one aspect less addressed throughout marketing professionals and communication efforts. Our results demonstrate the significance of emotional arousal on blood donation intentions, thus suggesting a more emotionally resonant approach of attracting potential donors.

## 1. Introduction

According to the most recent empirical data, the worldwide demand for blood is steadily increasing, indicating an urgent need to reinforce and scientifically improve the processes for recruiting and retaining blood donors [1,2,3]. In the social sciences, research has generally concentrated on connecting with and recruiting new blood donors, whereas research efforts in the behavioral sciences have primarily focused on preserving the current blood donor base [4,5]. Despite their complementary nature, these two scientific methodologies have been used independently up to this point. However, the theoretical urge for thorough unification and synergy between these two paths grows stronger, with the goal of creating and implementing more effective blood donation methods [4,6,7].

The primary emphasis of behavioral sciences research has been to examine the implications that negative experiences during blood donation could have on future blood donations [2,3]. Furthermore, it studies how various behavioral intervention strategies could potentially be utilized to retain existing blood donors. Understanding the motives that underlie voluntary blood donation is critical for improving the success of donor recruiting and retention initiatives, according to a study conducted by Glynn et al. [8]. Much of the relevant literature has demonstrated that altruism and prosocial dispositions are the primary motivators for blood donation [7,8,9]. Aside from altruism, other important contributing factors in the blood donation process include the awareness of the urgent need for blood, the influence of social factors, the desire to provide for relatives or friends, and the boost to personal self-esteem accompanied by social recognition. Researchers researched and recorded these driving elements in academic studies [10,11,12,13].

Extrinsic motivational factors, such as influence from social relationships with friends or family, as well as incentive through monetary rewards and gifts, have, instead, been identified as significant motivators for initial participation in blood donation, according to research [14,15,16]. These external motivations eventually decrease in importance as volunteer blood donors solidify their blood donor identity and form a more stable and inwardly driven role. This process becomes visible after the third or fourth repeated blood donation, as evidenced by the research of [2,6,8].

The formation of particular behavioral characteristics and outcomes is a multifaceted phenomenon involving the intricate interaction of cognitive processes, learning mechanisms, prevalent attitudes, and a variety of motivating elements. To gain an exhaustive and detailed understanding of the behavior of volunteer blood donors, researchers have explored a wide range of factors. The theory of planned behavior (TPB) constitutes a reliable and robust framework of behavior that culminates in a definitive outcome. It was developed by Ajzen and Madden [17], Ajzen [18] as an extension of the theory of reasoned action [19,20], with the objective of overcoming the original model’s limits in processing behaviors where volition is insufficient for full control. The TPB has achieved recognition as an exceedingly valuable tool for defining, quantifying, and experimentally analyzing the aspects that impact both the behavioral and prospective dimensions. It is based on the premise that the intention to adopt a behavior is the primary path to its fulfilment, with the assumption that the stronger the intended motivation in question, the higher the prospects for the successful execution of the desired behavior. According to TPB, the primary factor preceding and mediating behavior intentions is the drive to engage in it (actual behavior), which is predicted by three major variables: (a) individual attitude towards the specific behavior (attitude), (b) internalized subjective social norms (subjective norms), and (c) perceived controllability of behavior (perceived behavioral control) [18,19,21].

One particular aspect that could interfere with the logical decision-making process and TPB does not include, is that of emotional reactions and blood donation anxiety [21,22,23]. In behavioral contexts such as blood donation, where there is substantial variation between extensive cognitive assessments and emotional responses, the decision to donate blood could involve a number of deeply emotionally charged beliefs and implications that are not covered by the aforementioned theory. Such emotional reactions, especially in situations where the consequences of a behavior could have counterproductive and life-changing consequences, emotions can act as critical influencing factors in shaping individual attitudes and intentions [20,24,25].

The objective of this study is to implement a theoretical framework based on TPB to identify the psychosocial aspects that influence an individual’s intentions to donate blood. For this purpose, we designed ads that encourage and invite citizens to donate blood. Each advertisement comprises a core picture element intended to provoke an identifiable emotional response, as well as a textual narrative that highlights a reason for participating in blood donation. We aimed to investigate the mediating role of emotional arousal from the ads on future behavioral intention to donate blood and its influence on the participants’ motives, acknowledging the significance of advertising messages in volunteering behavior in the context of blood donation. Additionally, we expand our theoretical model beyond the standard TPB constructs with the inclusion of blood donation anxiety, attitude towards advertisements, and emotional arousal (positive/negative) to provide a comprehensive overview of both attitudinal and behavioral influences that could potentially affect blood donation behavior.

Health professionals have always been at the forefront of recruitment strategies that meet the urgent need for blood and organ donors. Nevertheless, this tactic often neglects the possibility of emotional arousal in marketing implementations, which is normally the preserve of marketers. Utilizing structural equation modeling as a source of understanding donor behavior, this study aims to bridge this gap and shed light on this issue, by investigating the mediating role imposed by emotional arousal and blood donation anxiety on intentions to donate. Thus, our research contributes to a reorientation in recruitment campaigns that are more compassionate, empathetic, and centered on donors by examining emotional behavior and knowledge among potential donors.

The article is structured as follows: Section 2 provides the relevant literature review on the current topics and determinants that influence blood donation behavior. In addition, we place particular emphasis on the factors of donation anxiety, attitude towards advertisements, and the impact of the theory of planned behavior in the context of blood donation. Moreover, we place particular emphasis on the role of emotional arousal with regard to future intentions to donate blood. In Section 3, we provide a detailed description of the model we constructed and evaluated in our research, followed by the analysis and presentation of our findings in Section 4. Section 5 discusses and interprets the results of our study and identifies related limitations. Finally, we conclude and provide suggestions for future research.

## 2. Literature Review

### 2.1. Determinants of Blood Donation Intentions

Smith’s altruistic approach recognizes humanitarianism as an essential incentive for voluntary blood donation. However, Rapport and Maggs [26] pointed out that altruism is not the sole justification for performing unpaid blood donations. He underlined the difficulties of describing any type of blood donor as absolutely selfless and entirely spontaneously altruistic. The need for a deeper sense of responsibility, gratitude, and self-interest, as well as a more conscious awareness about the significance and intended use of given blood, were emphasized. In the context of the research on the subject of voluntary blood donation, empirical studies indicate that the relevant behavior can be influenced by both altruistic and egocentric motivations [27,28,29]. Additionally, as human interactions and collaboration have been associated with the activation of reward mechanisms [2], blood donation appears to be influenced in part by the expectation of personal satisfaction combined with the desire to provide and assist others [2,4,5]. Parash, Suki, Shimmi, Hossain and Murthy [24] investigate the behavioral intention of students in Malaysia to voluntarily donate blood, utilizing the theory of planned behavior (TPB) as a guiding framework. The results indicate that knowledge plays a crucial role in influencing students’ intention to make voluntary blood donations. Specifically, a strong correlation was found between students’ knowledge about the impact of blood donation on saving lives and their inclination to donate blood. Another approach is that of Allah Pitchay et al. [30], who investigate the factors influencing individuals’ intentions to participate in donation crowdfunding, utilizing the self-determination theory. Specifically, a sense of self-worth, perceived donor effectiveness, and moral obligation were identified as significant drivers of DI. Also, there is an effort in the academic community to provide recommendations for social marketing designers to enhance the retention of blood donors. Van Steenburg and Spears [31] investigate how individuals respond to donation messages in broadcast advertising, considering both preexisting attitudes and beliefs related to donating and message processing.

By understanding the interplay between motivational factors and established behavioral theories, the study of M’Sallem [25] offers insights that can guide strategies to address the issue of unsustainable blood donations and contribute to more effective blood donation campaigns. The significance of the quality of the donation process in shaping donor satisfaction and strengthening both donor trust and loyalty is discussed by Alzola-Melian et al., 2020.

Chell and Mortimer [32] links the dimensions of donor value (altruistic, emotional, and social) and conspicuous donation behavior. The findings contribute valuable insights into the usage of conspicuous donation tokens of recognition on social media and enhance our understanding of the under-researched areas of donor value and conspicuous donation behavior. Williams et al. [33] aimed to test a model that integrates self-determination theory (SDT) and the theory of planned behavior (TPB) to predict the intention to donate blood. This research demonstrates that integrating SDT and TPB is a useful approach in blood donor research, providing evidence of plausible pathways through which motivational orientations impact the intention to donate blood. In another attempt, Torrent-Sellens et al. [34] identified three main prediction paths. The first two originate from feelings of trust in the digital community and a positive mood associated with a modern lifestyle. These paths are linked to attitudes and behavioral control, contributing to the explanation of both the intention to donate and the actual act of blood donation. The third path originates from modern lifestyles and is connected to the subjective norm, predicting both intention and actual donation.

Griffin, Grace and O’Cass [16] aim to enhance the understanding of donors and non-donors by exploring factors such as social responsibility, susceptibility to interpersonal influence, involvement in and attitude towards blood donation, and aroused feelings. In a similar matter, Bartsch and Kloß [35] investigate the impact of personalized charity advertising on promoting empathy, attitude change, and intentions to help stigmatized social groups. The study highlights the dual nature of personalized charity advertising: while it enhances involvement, empathy, and prosocial outcomes, it also triggers reactance. Pentecost et al. [36] suggest that emphasizing positive emotional feelings and the sense of control in the act of donating blood may be a more effective strategy than using external regulation, which implies a sense of obligation and may limit blood donation numbers. Another suggestion are the charity gift cards (CGCs), allowing recipients to allocate their gift across various charitable projects, that Mulder and Joireman [37] propose in their paper. Another significant finding is that from the survey of Chen et al. [38], which indicates that the negative association between blood donation anxiety and intention is mediated by moral disengagement. Furthermore, mindfulness was identified as a buffering factor, playing a role in mitigating the relationship between blood donation anxiety and moral disengagement, as well as the indirect relationship between blood donation anxiety and intention through moral disengagement.

### 2.2. Theory of Planed Behavior and Blood Donation

Numerous theoretical models have been developed to provide practical guidance and solutions to better understand and address the issue and lack of enough blood supplies. Among these, the theory of planned behavior, as described by Hardeman et al. [39], appears to be a potential approach for addressing this issue. This theory has been widely applied in empirical research to predict a variety of health-related behaviors. Particular emphasis has been placed on adopting healthy habits, performing preventative medical exams, and adhering to treatment plans [23,24,33,40,41,42,43]. Finally, it has been demonstrated that the theory helps to the prevention of cancer and the promotion of voluntary blood donation [24,25,42,44].

In our study, we primarily employ the theory of planned behavior (TPB) as the foundation to better understand the factors that influence blood donation intentions. Based on TPB, we further expand our conceptual framework by introducing elements of the attitude towards advertisements (AAD), blood donation anxiety (BDA), and further extended by studying the impact of emotional arousal. This broadened methodology provides an enhanced comprehension of the processes that govern the decision of providing blood willingly. This research process and strategy enable us to investigate a wider range of factors that influence and affect blood donation intentions. Personal views have an important role in determining individual attitudes towards various types of behavior in the field of behavioral science, with impacts ranging from positive to negative [4,17,21,45]. These attitudes are developed by attentively experiencing the predicted outcomes of engaging in a given behavior, as well as the evaluations that follow from those results. Thus, people’s behaviors could vary depending on the dominant attitudes they have developed.

However, the existing academic literature reveals a relative lack of empirical studies investigating the relationship between elements of TPB and various dimensions of anxiety in the field of voluntary blood donation, such as the degree and frequency of anxiety levels, panic episodes, and the effect of anxiety on the final decision to donate blood [20,21,24,25,39]. Thus, applying and expanding this theory with different behavioral and attitudinal constructs can be an essential procedural tool for decision-makers in the field of strategy and health policy development. In current research incorporating TPB into blood donation, pooled samples of individuals, including active and non-donors, are utilized with the aim of analyzing blood donation decision-making depending on the donors’ blood donation phase. This approach focuses on the development of the “temporal trajectories” of donors, examining how novice donors become regulars [2,3,4]. Ferguson [15] points out the existing inconsistency regarding the classification and definition of blood donors at different stages of their journey, while recognizing the importance of how the theoretical approach should adjust for newcomers and experienced blood donors. According to Masser et al. [46], the integration of various constructs into the research process includes the analysis of moral norm, anticipated regret, past behavior or habit, self-identity, and emotional reactions in order to explore the transition from casual to regular blood donors and their influence in the decision-making process.

### 2.3. Emotional Appeals and Anxiety in the Context of Blood Donation

A common and significant critique levelled against TPB is the apparent absence of emotional components from the process of understanding and forecasting behavior [19,21,23,24,25,47]. Proponents of the theory argue that while emotive attributes could not be expressed explicitly, they are implicitly assimilated through subjective norms and perceived behavioral control. At the same time, as Ajzen and Fishbein [19], Ajzen [22] point out, the addition of the aspect of emotional implications from executing a behavior does not appear to have a significant effect on the predictability associated with that behavior. However, it has been shown that people in a positive affective state perceive positive events (such as the consequences of a given behavior) as those that they are more likely to adopt, and vice versa, which suggests a strong influence in shaping both the intention and eventual adoption of a behavior [40,41,45,48]. Telle and Pfister [49] focus on the relationship between empathy, particularly for positive emotions, and prosocial behavior. While existing research has extensively studied empathy in conjunction with negative emotions, there is a gap in understanding the connection between positive empathy and demonstrated prosocial behavior. The article proposes that individuals’ motivation to maintain a positive emotional state serves as a key mechanism linking positive empathy to prosocial behavior. One very important view on the subject of donation and charity is the recent one of Bae [50]. Their study examines the impact of message order on recipients’ responses to charity advertisements using the expectancy-contrast and negative-state relief models. The results show that a negative–positive message order evokes positive emotion, while a positive–negative order generates negative emotions. Both positive and negative emotions induced by charity advertisements affect helping intention through positive and negative empathy. This study enhances the current understanding of the roles of emotion and empathy in charity intention formation. On the same topic, Homer [51] examined the effectiveness of emotional appeals, proposing a sympathy and inspiration-helping hypothesis. The results suggest that combining hope and sadness in appeals generates both sympathy and inspiration, leading to increased donations for human-suffering causes. Additionally, improving emotion appeals that shift from sad to hopeful is found to result in higher donations compared to declining emotion appeals that shift from hopeful to sad. Similarly, Bennett [52] showed that mixed emotions play a significant role in shaping both the attitude towards the advertisement and participants’ intentions of future donations. Nabi [53] examines the persuasive influence of emotions in health messages, emphasizing a shift from focusing on individual emotions to exploring the flow or evolution of emotional experiences during exposure to a message. Morelli et al. [54] address the three limitations of empathy, affective congruence, perspective-taking, and prosocial motivation, by assessing empathic responses to individuals experiencing various emotions with variations in valence and social context. Functional magnetic resonance imaging reveals that empathy for positive and negative emotions selectively activates regions associated with the corresponding affective states.

Another subject in the context of blood donation is how anxiety acts as a disincentive and influences donors’ behavioral intentions. Overall, in many cases, anxiety plays a key role in the psychological situation of the patient [55,56]. The study of Chen, Zhou, Zhang and Xiao [38] aimed to construct a model outlining the relationship between blood donation anxiety and intention. Their results revealed that the adverse association between blood donation anxiety and intention was mediated by moral disengagement. Additionally, mindfulness emerged as a key factor buffering the link between blood donation anxiety and both moral disengagement and the indirect relationship between anxiety and intention through moral disengagement. In comparison, Hoogerwerf et al. [57], Hoogerwerf et al. [58] indicated that in individuals who experienced negative incidents during their initial blood donation, there was no significant correlation between anxiety levels and their attitude toward blood donation.

In a different approach, in the study of Robinson et al. [59], the authors used as measurements the standard theory of planned behavior and, additionally, they incorporated measures for descriptive norm, moral norm, donation anxiety, and anticipated regret. Through path analysis, they evaluated the effectiveness of an augmented TPB model in predicting the intentions of 195 individuals who had not previously donated blood, and found that perceived behavioral control, moral norm, descriptive norm, anticipated regret, and donation anxiety are direct predictors of intention. Moreover, moral norms, donation anxiety, and donor identity indirectly influenced intention through attitude [44]. In a case study in Pakistan, researchers used the augmented theory of planned behavior (TPB) model and revealed key pathways in understanding the relationship between blood intention and behavior [42]. Their model highlighted that moral norm, donation anxiety, and donor identity had indirect effects on intention through their influence on attitude. On the other hand, some studies underline the critical role of the blood banks and hospital services in the process of blood donation and how they can improve it [60,61]. Bednall, Bove, Cheetham and Murray [4], Bednall and Bove [7] emphasized the importance of improving donors’ attitudes, enhancing perceived behavioral control and self-efficacy, and minimizing the risk of adverse reactions. Implementing re-recruitment policies for temporarily deferred donors is also recommended to safeguard future donation behavior. The findings have implications for blood collection agencies and researchers in enhancing blood donation practices [4]. Significant results arise from the study of France et al. [62], regarding the relationship between anxiety and blood donation. The overall consequence was a further decrease in donation intention. In essence, the study highlighted the multifaceted influence of anxiety on various aspects of the blood donation experience and its subsequent impact on donors’ intentions to contribute again. Studies have shown that anxiety has a serious effect on the cognitive tasks [63].

## 3. Research Methodology

### 3.1. Methodology and Measurements

**Figure 1:**
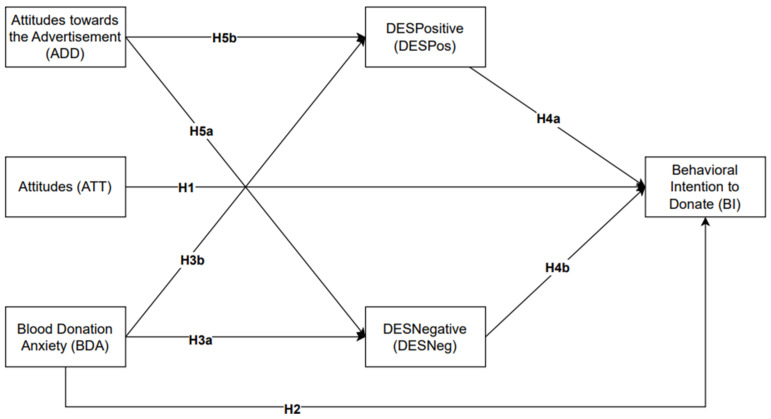
Conceptual research model.

The purpose of this research is to develop a conceptual framework that can be used to analyze the complex motivating factors involved in voluntary blood donation. It aims to combine constructs of the TPB such as emotional arousal, attitudes, attitudes towards the advertisement, blood donation anxiety, and behavioral intention provided. The refined process captures the core idea of altruistic behavior, which makes blood donations distinct and signify the pivotal roles in forming prosocial and altruistic behaviors that are inherited in the act of blood donation. With the inclusion of emotional arousal, either positive or negative, and blood donation anxiety, our efforts provide useful insights into the emotional dimension the facilitates the donation process, while attitudes reflect on the accumulated beliefs and people’s predispositions. We examine the potential outcomes that derive from adapting attitudes and emotional inclinations into behavioral intention, that is captured within the TPB framework, as a direct relation to action. Our research attempts to understand human nature and its complexity within the psychological landscape that surrounds blood donation, and to offer a wide-ranging analysis of the various elements that have an impactful role in the context of voluntary blood donation. This research approach employs quantitative data collection methods and seeks to uncover the interrelations of these factors and enrich the public health and voluntary blood donation practices. A visual representation of this research model is provided in Figure 1.

To address this gap between theoretical motivations and practical intentions, a tailored quantitative analysis was performed, in particular, a quantitative non-experimental correlation design adapted from [64,65]. An explicit self-administrated web survey was conducted through particular sites typically used by potential and existing blood donors. This decision deliberately reflects the peculiarities environing blood donation towards ensuring a comprehensive understanding of factors that motivate or hinder this crucial act.

We employed an online survey as our primary data collection tool, taking advantage of multiple channels to engage a wide cross-section of participants, encompassing academia and the wider Greek populace. Our questionnaire of 20 questions (Appendix A) emphasized the essence of voluntariness while acknowledging the sensitivity surrounding blood donation. We therefore went beyond traditional motivations and ethical considerations since our investigation probed inherent inspirations as well as moral concerns regarding the momentousness of this sort of donation on both parties involved in it—giver and recipient alike. This approach aimed to increase variety in the sample population, hence improving the representativeness and validity of the findings.

### 3.2. Experimental Setup

Engagement in this study was purely on a volunteer basis, with individuals encouraged to share their insights on blood donation from a personal viewpoint. The current study employed an experimental design to investigate the extent to which emotional arousal and other motivating factors impact the desire to donate blood voluntarily. Two sets of three advertisements were devised, separated by their emotional tone (positive vs. negative), whilst each message was presented via two prisms, namely, altruistic and egocentric textual messages, resulting in a collection of twelve ads. The research process involved assigning individuals to each of the advertising conditions and documenting their emotions and intents to donate blood following exposure to the advertising messages.

It is important to clarify that we conducted a pre-test as an online survey to ensure that the stimuli that would be used in the experiment actually evoked the desired emotions. We placed particular emphasis on how diverse emotions lead to voluntary blood donation. A focal point of our research is to examine the effects of modern marketing strategies on the advertising of voluntary blood donation, and how they can boost credibility and interaction with potential volunteers, with the inclusion of elicited emotional responses, either positive or negative. This approach attempts to promote emotions of interest, inspiration, and joy, while also introducing negative feelings that could lead to discouragement or distrust, such as disgust, guilt, and fear, which were investigated in the context of our study. As far as the textual messages are concerned, as mentioned above, we examined the altruistic and egoistic motives, with which we aimed to understand how different types of motivations influence the behavioral intentions of the respondents. The altruistic version of the ads included messages such as “Have you considered the possibility that one of your close relatives needs blood urgently?” and “Is a small and quick pinch so important... that you refuse to save the lives of three people?”, whilst egoistic messages included the following: “Volunteer blood donors have priority in case they need blood” and “Donating blood can increase the life span of the donor”. The objective was to improve the effectiveness of advertising campaigns whilst avoiding potential biases and favoritism towards certain popular blood donation services. A cumulated presentation of the ads is depicted in Table 1. A preliminary online survey was administered to ensure that the experimental stimuli evoked appropriate emotional responses within the context of voluntary blood donation. Although message framings were conceptually implemented in our research design, we did not account for their moderating effects in order to be included as part of future studies, and directed our efforts towards examining the mediating roles of emotional arousal as the primary scope of this research.

To enhance participation in the survey, various distribution and sample gathering methods were applied, such as snowball sampling [66], encouraging participants to spread the questionnaire to their social network (friends, family, etc.) after completing it. As an incentive to increase participation, the option to enter a raffle for one-hundred-euro gift vouchers was incorporated to motivate participants during questionnaire completion.

### 3.3. Research Framework, Instruments, and Metrics

In the context of this research, we employed measuring methods based on previous scientific studies and added new and innovative elements in our approach which was organized by demographic and scale measurements. Demographic statistics were collected to obtain basic information about the sample, which could potentially be utilized as control variables in future analyses to further segment and assess various groups. In the second phase of the study, participants were presented with selected advertising messages, followed by a post-experiment questionnaire. The second part was devised to evaluate the effectiveness of ads in terms of achieving emotional or logical connection to the audience, as well as the emotional arousal utilized. Based on their exposure to the ads, participants answered a series of questions to measure behavioral and attitudinal changes.

Specifically, the blood donation anxiety (BDA) construct was adapted from Chen, Zhou, Zhang and Xiao [38] and Robinson, Masser, White, Hyde and Terry [38,59], and measured on a three-item scale. The scale includes the following statements: “In the future if I donate blood, I would feel nervous”, “In the future, if I donate blood, I would feel distressed”, and “In the future if I donate blood, I would feel anxious.”. Attitudes (ATT) were adapted and measured with a semantic differential scale from the TPB theoretical model [18,19]. The construct included items of satisfaction from blood donation (unsatisfying/satisfying), meaningfulness (pointless/worthwhile), sense of fulfillment (unrewarding/rewarding), and emotional experience (stressful/relaxing). Attitudes towards the advertisement were measured with a five-item semantic differential scale obtained and adapted from Holbrook and Batra [67] and Ranganathan and Henley [68]. Emotional arousal was measured with the Greek version of the Differential Emotion Scale (DES), which distinguishes between positive and negative emotional states [69]. DESPos describes positive feelings (joy, inspiration, and interest), whereas DESNeg refers to negative emotions such as guilt, disgust, and fear. The scale was used to verify the success in capturing the necessary emotions. Finally, blood donation intention (BI) was measured with a three-item scale adapted from the TPB theoretical model [18,19,22]. It incorporated items that evaluated the likelihood of engaging in blood donation in the near future (“I intend to donate blood in the next 3 months”, “I will donate blood in the next 3 months”, and “I would like to donate in the next 3 months”).

Thus, this scalar approach allows for an in-depth understanding of the factors that influence voluntary blood donation intentions and formed hypotheses to capture and evaluate the relationships between these factors. The hypotheses evaluated and analyzed in our structural equation model are listed below:

**H1.**  *Attitudes (ATT) directly influence the behavioral intention to donate (BI).*

**H2.**  *Blood donation anxiety (BDA) has a direct negative influence on the behavioral intention to donate (BI).*

**H3a.**  *Blood donation anxiety (BDA) has a direct effect on DESNegative (DESNeg).*

**H3b.**  *Blood donation anxiety (BDA) has a direct effect on DESPositive (DESPos).*

**H4a.**  *DESPositive (DESPos) directly influences the behavioral intention to donate (BI).*

**H4b.**  *DESNegative (DESNeg) has a direct negative influence on the behavioral intention to donate (BI).*

**H5a.**  *Attitudes towards the advertisement (ADD) have a direct effect on DESPositive (DESPos).*

**H5b.**  *Attitudes towards the advertisement (ADD) have a direct effect on DESNegative (DESNeg).*

**H6a.**  *The link between attitudes towards the advertisement (ADD) and the behavioral intention to donate (BI) is mediated by the positive emotional arousal (DESPos).*

**H6b.**  *The link between attitudes towards the advertisement (ADD) and the behavioral intention to donate (BI) is negatively mediated by the negative emotional arousal (DESNeg).*

**H7a.**  *The link between blood donation anxiety (BDA) and the behavioral intention to donate (BI) is mediated by the positive emotional arousal (DESPos).*

**H7b.**  *The link between blood donation anxiety (BDA) and the behavioral intention to donate (BI) is negatively mediated by the negative emotional arousal (DESNeg).*

Variables were measured using a five-point Likert scale, meticulously adapted, and translated to maintain question integrity and ensure statistical validity for Greek respondents, under the guidance of a multilingual scientific advisor. Following a preliminary review to address any phrasing inconsistencies, data from the pilot testing were excluded from the final analysis.

### 3.4. Sample Profile

The data collection received a total of 414 replies. In terms of gender, participants were nearly evenly split, with 52.2% identifying as male and 47.8% as female. The age distribution of respondents was biased towards younger individuals, with 30.0% aged 18–25, 25.8% aged 26–30, and the remaining in older age groups. In terms of educational qualifications, 5.6% of the sample were high school graduates, 33.8% were undergraduates, 28.0% had graduated, 17.1% were postgraduates, 11.6% were postgraduate students, 2.9% were PhD candidates, and 1.0% possessed doctoral degrees. Table 2 summarizes the demographic details.

## 4. Data Analysis and Results

In the current study, data were analyzed using the Smart-PLS4 program (version 4.1.0.0), with structural equation modelling (SEM) serving as the primary analytical tool. This approach is well recognized for its robustness in variance-based SEM applications in management and social sciences, as shown in the work of Nitzl et al. [70]. Furthermore, the current research employed partial least squares structural equation modelling (PLS-SEM) because of its proficiency in causal modelling and focus on optimizing the explained variance in dependent latent variables. To ensure the precision of beta coefficients, reliability indices, and standard errors, the methodological standards established by Wong [71] were strictly followed. This included validating that all indicators were properly aligned with their respective latent constructs and that outer loadings surpassed the 0.7 criterion when assessing the reflective measurement model.

### 4.1. Measurement Model

The initial phase of the PLS-SEM process includes the assessment of the measurement model. In this framework, the model’s constructs were quantitatively analyzed utilizing reflective measures, which included tests of composite reliability, indicator reliability, convergent validity, and discriminant validity, as defined by Hair et al. [72]. The initial evaluation of the measurement model begins with an analysis of indicator reliability to determine the amount to which a variable’s variance is accounted for by the construct it is intended to assess, as explained by Chin [73]. These measurements are represented in the size of outer loadings, as defined by Wong [71], which should ideally be less than 0.70, according to Chin [74]. Vinzi et al. [75] acknowledge that while factor loadings greater than 0.7 are generally favored, social science research frequently produces lower outer loadings (less than 0.70). The choice of whether to maintain or reject items with poor loadings requires a thorough assessment of their impact on composite reliability and convergent validity, rather than premature deletion. Indicators with outer loadings ranging from 0.40 to 0.70 may be removed only if they improve the construct’s composite reliability or average variance extracted (AVE) beyond the acceptable threshold, as established by Hair et al. [76]. Table 3 shows that the refining of the measurement model resulted in the deletion of two items (ATT4 and AAD5) owing to inadequate factor loading (<0.500), which is consistent with the criteria set by Gefen and Straub [77].

In the present investigation, reliability was assessed using Cronbach’s alpha, rho_A, and composite reliability as evaluation indices. All parameters surpassed the baseline tolerance limit of 0.700, as determined by Wasko and Faraj [78]. The rho_A statistic, which is conceptually positioned between Cronbach’s alpha and composite reliability after the discussion of Sarstedt et al. [79], was similarly above the 0.7 barrier, hence substantiating the robustness of reliability, as confirmed by Henseler et al. [80]. Furthermore, convergent validity was confirmed to be acceptable, with the average variance extracted (AVE) being above the 0.500 criterion in the majority of cases, consistent with Fornell and Larcker’s standards [81]. Discriminant validity was evaluated by comparing inter-construct correlations to the square root of the AVE, as described by Fornell and Larcker [81], and through the heterotrait–monotrait (HTMT) correlation ratio, as proposed by Henseler, Hubona and Ray [80]. The resulting values were less than the rigorous cut-off of 0.85, indicating discriminant validity, as shown in Table 4 and Table 5.

### 4.2. Structural Model

As outlined by Hair Jr et al. [82], the structural model was assessed within the designated research framework using R^2^, Q^2^ values, and the path coefficients’ significant levels. In this study, the R^2^ values, a measure of the variance explained by the model, ranged from 0.395 for behavioral intention to donate to 0.210 for DESNegative and 0.339 for DESPositive, confirming their positioning within the expected range of 0 to 1. The suggested model was shown to be predictively effective, with Q^2^ values of 0.333 for behavioral intention to donate, 0.199 for DESNegative, and 0.326 for DESPositive. The model’s robustness was further confirmed using hypothesis testing, which determined the significance of the relationships between constructs. The bootstrapping approach was employed to examine path coefficients and their statistical significance, following the guidelines outlined by Sarstedt, Ringle and Hair [79]. This study also included the mediation analysis methodologies proposed by Preacher and Hayes [83], and, in accordance with Streukens and Leroi-Werelds [84], applied a bootstrap sample size of 10,000. Table 6 presents the empirical data from these investigations.

The results indicated a significant impact of attitudes (ATT) on behavioral intention to donate (BI) (β = 0.084, *t* = 2.173, *p* < 0.05), thereby supporting H1. Furthermore, blood donation anxiety (BDA) was found to influence behavioral intention to donate (BI) significantly and positively (β = 0.261, *t* = 4.959, *p* < 0.001), which partially supports H2. The analysis also revealed that BDA has a significant effect on DESNegative (DESNeg) (β = 0.287, *t* = 4.705, *p* < 0.001), supporting H3a, and a significant effect on DESPositive (DESPos) (β = 0.234, *t* = 4.586, *p* < 0.001), supporting H3b. Additionally, the DESNegative (DESNeg) was shown to significantly and positively affect behavioral intention to donate (BI) (β = 0.303, *t* = 6.515, *p* < 0.001), partially confirming H4b, while DESPositive (DESPos) also significantly influenced behavioral intention to donate (BI) (β = 0.226, *t* = 4.766, *p* < 0.001), hence H4a was supported. Attitudes towards the advertisement (AAD) had a significant impact on DESNegative (DESNeg) (β = 0.227, *t* = 4.060, *p* < 0.001), supporting H5a. Moreover, AAD was significantly related to DESPositive (DESPos) (β = 0.413, *t* = 8.308, *p* < 0.001), which supports H5b. The empirical evidence from these tests is consolidated in Table 5.

#### Mediation Analysis

Mediation analysis was conducted to assess the mediating effects of emotional responses on the relationships between attitudes and behavioral intentions. The results revealed that the direct effect of blood donation anxiety (BDA) on behavioral intention to donate (BI) was significant (β = 0.261, *t* = 4.959, *p* < 0.001). Similarly, the direct effect of attitudes towards the advertisement (ADD) on behavioral intention to donate (BI) was significant (β = 0.344, *t* = 7.163, *p* < 0.001). The total effect of attitudes towards the advertisement (AAD) on behavioral intention to donate (BI) was significant (β = 0.162, *t* = 5.234, *p* < 0.001), and the total effect of blood donation anxiety (BDA) on behavioral intention to donate (BI) was also significant (β = 0.401, *t* = 8.878, *p* < 0.001). Regarding specific indirect effects, the indirect effect of AAD on BI through DESPositive (DESPos) was significant (β = 0.093, *t* = 3.630, *p* < 0.001), supporting H6a. The indirect effect of AAD on BI through DESNegative (DESNeg) was also significant (β = 0.069, *t* = 3.094, *p* = 0.002), partially supporting H6b, as we hypothesized a negative relationship. Additionally, the indirect effect of BDA on BI through DESPositive (DESPos) was significant (β = 0.053, *t* = 3.496, *p* < 0.001), supporting H7a, and the indirect effect of BDA on BI through DESNegative (DESNeg) was also significant (β = 0.087, *t* = 4.093, *p* < 0.001), thus H7b was partially supported. These findings indicate the presence of significant mediating effects of emotional responses in the relationship between attitudes, anxiety, and the intention to donate blood.

The mediation analysis revealed partial mediation in the relationships examined. Although the direct effects of blood donation anxiety (BDA) on behavioral intention to donate (BI) (β = 0.261, *t* = 4.959, *p* < 0.001) and attitudes towards the advertisement (ADD) on BI (β = 0.344, *t* = 7.163, *p* < 0.001) were significant, the presence of significant indirect effects through both DESPositive (DESPos) and DESNegative (DESNeg) demonstrates the mediating role of these emotional responses. Specifically, the indirect effects of AAD on BI through DESPos (β = 0.093, *t* = 3.630, *p* < 0.001) and DESNeg (β = 0.069, *t* = 3.094, *p* = 0.002), as well as the indirect effects of BDA on BI through DESPos (β = 0.053, *t* = 3.496, *p* < 0.001) and DESNeg (β = 0.087, *t* = 4.093, *p* < 0.001), were found to be statistically significant, confirming the partial mediating effect. Therefore, while the emotional responses mediated the relationship between the independent variables and the intention to donate blood, they did not fully account for the observed effects, indicating that other factors directly influenced the behavioral intention too. The detailed results are displayed in Table 7.

## 5. Discussion

In our study we employed a quantitative non-experimental correlation design and a SEM analysis approach to investigate the varying factors that impact blood donation intentions. Considering both behavioral and attitudinal influences, we included in our research the key elements of behavioral intentions, blood donation anxiety, attitudes, emotional arousal, and attitudes towards advertisements supporting blood donation campaigns. Our investigation revealed intriguing relationships between the variables in question.

The analysis of direct effects within the study highlights critical insights into the factors influencing the intention to donate blood. The significant direct effect of blood donation anxiety (BDA) on behavioral intention to donate (BI) highlights the influence of personal anxiety in the decision to donate blood, supporting hypothesis H2. Attitudes towards the advertisement (ADD) also have a direct and significant impact on BI, confirming H5b and underscoring the effectiveness of advertisement content in influencing donation intentions. The confirmed significant relationship between general attitudes (ATT) and BI, as indicated by H1, suggests that intrinsic beliefs and perceptions about blood donation are closely linked with the actual decision to engage in donation behavior. These direct relationships emphasize the need for strategies that mitigate donation-related anxieties and leverage the persuasive impact of advertisements to boost blood donation rates. Findings concerning the direct effects of blood donation-related stress (BDA) on negative (DESNeg) and positive (DESPos) emotional arousal, as captured in hypotheses H3a and H3b, demonstrate the privileged position of stress as an important affective influencer in blood donation. Subsequently, the analysis revealed that both emotional states, negative (DESNeg) and positive (DESPos), play a critical role in behavioral intentions, as supported by hypotheses H4a and H4b, highlighting the influence of emotional states on the shaping of behavior. Additionally, the direct correlation of a positive attitude toward advertising (ADD) with a positive emotional state (DESPos), as stated in hypothesis H5a, suggests the decisive contribution of positive emotional stimuli resulting from advertising efforts. Overall, the study findings draw attention to the need for targeted interventions that address stress and enhance positive emotional experiences, with the goal of improving voluntary participation in blood donation. Placing particular emphasis on the effect of emotional arousal and attitudes toward advertising on behavioral intentions, the need for more sophisticated communication strategies aimed at combating negative emotions and promoting positive emotional motivation becomes apparent. In particular, positive emotional activation plays a central role as a catalyst, encouraging willingness to donate blood, minimizing the adverse effects of stress, and exploiting the positive effects of promotional efforts. Correspondingly, negative affective activation points to the risk that adverse affective reactions can divert the intention to donate, despite the existence of favorable attitudes or persuasive advertising content. The dual nature of emotional mediation reveals the complexity in the decision-making process regarding blood donation, pointing out the necessity of effective management of emotional responses to enhance efforts to attract and retain blood donors.

Thus, we further explored the potential impact of emotional arousal as a mediating factor. The mediation analysis revealed that emotional arousal, specifically positive (DESPos) and negative (DESNeg), is identified as a mediating factor in the effect of attitudes toward advertisements (ADD) on behavioral donation intention (BI), validating hypotheses H6a and H6b. This partial mediation through affective reactions indicates that although emotions evoked by advertisements have a role in determining donation intentions, they are not the only factor shaping the overall influence of individuals’ attitudes toward the due to advertisements. Additionally, the indirect effect of blood donation stress (BDA) on BI through emotional reactions, as evidenced by hypotheses H7a and H7b, demonstrates the multidimensional contribution of stress, both directly and indirectly, through the activation of emotional reactions that affect the decision-making process of donating blood. The observed partial mediation points to the need for the implementation of holistic promotional strategies, which will consider both emotional and rational factors that shape individuals’ intentions towards voluntary blood donation. This approach enhances the understanding of the complex nature of the decision-making process to donate blood and highlights the importance of providing multidimensional information and awareness campaigns in the context of blood donation. Furthermore, incorporating the findings into action planning can optimize the effectiveness of blood donation promotion initiatives, increasing the attraction of new donors and enhancing repeat donation from existing ones.

To conclude, our research provides an in-depth analysis of blood donation intentions whilst aligning with and extending the current literature. By including emotional arousal, both positive and negative, within the framework of the theory of planned behavior (TPB), this study notably contributes to an improved understanding of the effect of emotional aspects on blood donor intentions. This addition provides a more thorough model of analysis that represents the donor’s broader behavioral dynamics, bridging a theoretical gap in the current literature that previously disregarded the emotional side [20,21,23,25]. Additional insights into how anxiety acts as a preventing factor to blood donation are revealed by this study’s singular analysis of anxiety related to blood donation, which examines both the direct and mediating impacts of anxiety on behavioral intentions [38,62,85]. This approach enhances TPB by emphasizing the importance of emotional states and broadening the set of criteria used for predicting and interpreting blood donation behavior. Furthermore, by empirically validating the influence of advertising on blood donation intentions, the study indicates the essential nature of focused communication strategies in the further development of the TPB model. The findings indicate the power of persuasive messages to change behavioral intentions, which contributes substantially to the larger discussion concerning the effectiveness of health communication efforts [50,86,87]. This study’s findings emphasize the crucial relevance of improving donor attitudes, attitudes, and self-efficacy, as well as shedding light on the effects of blood donation anxiety and emotional arousal. The necessity to employ more advanced and sophisticated methods and instruments for analyzing the behavior of blood donors is essential for obtaining an in-depth understanding of their psychology. Intervention strategies should be tailored to the different categories of blood donors, such as newcomers, occasional donors, and regular donors, with a focus on enhancing emotional incentive, such as promoting social interest and charitable giving, promoting cognitive empowerment through information about blood donation procedures, and negating false assumptions and fears associated with it [44,46,62,88].

Overall, our findings highlight the significance of addressing psychological factors such as blood donation anxiety and fostering positive attitudes towards both blood donation itself and related promotional materials. By targeting these variables, healthcare organizations and blood donation agencies can develop more impactful strategies to encourage and sustain blood donation behavior among the population.

## 6. Conclusions

Blood donation and strategies for donor recruitment represent critical concerns within a nation’s healthcare infrastructure. This study has unveiled the multidisciplinary nature of this issue, with insights from fields including psychology and marketing. The involvement of these disciplines underscores the profound trust donors and potential donors place in the blood donation process. The intersection of marketing with blood donation initiatives reveals insights into effective communication strategies and branding techniques. Through targeted messaging and branding efforts, organizations can enhance the public perception of blood donation, making it a more appealing and accessible option for potential donors. Moreover, it highlights their perception of donation as the pinnacle of altruism and societal contribution, devoid of any apprehension or anxiety. By harnessing the collective expertise of these fields, policymakers and healthcare professionals can develop more comprehensive and impactful strategies to address the ongoing need for blood donations in society.

In terms of broad ramifications and practical implications, this study sets the groundwork for policy development and program design to improve blood donation efficiency. Our findings about the direct and mediating functions of emotional activation provide important scientific perspectives for designing tactics that seek to increase donation activity. Establishing the effect of positive and negative emotional activations can lead to the development of more specialized advertising initiatives, aimed at attracting new donors and strengthening the commitment of existing ones. Furthermore, the emergence of anxiety about blood donation as a primary barrier to voluntary participation highlights the imperative to implement interventions focused on reducing donor anxiety. Building an environment that provides support to donors, as well as implementing communication strategies that dispel the most common misunderstandings and concerns associated with blood donation, can significantly increase the willingness to donate. Moreover, deepening the understanding of donation behavior allows for the development of cohesive strategies to overcome psychological, emotional, and informational barriers to voluntary contribution. In addition, combining elements of psychology and communication methods in the context of health behavior creates opportunities for interdisciplinary research. It encourages additional research into how various behavioral theories and communication frameworks could be utilized to understand and influence health-related behaviors. As a result, this study significantly contributes to the scientific discussion on blood donation by providing a reinforced theoretical framework and practical insights that could potentially be used to enhance donation performance and public health prospects. Finally, confirming the influence of advertising attitudes on blood donation intentions provides the foundation for the development of evidence-based marketing approaches within the field of blood donation. Organizations can take advantage of this information to create communications that resonate with the values and emotions of potential donors, thereby improving the effectiveness of their advertising campaigns.

This research is not without limitations. The findings of this research, which are based on the Greek context, may not be universally applicable due to differences in cultural and socioeconomic dynamics between regions. Future attempts could delve deeper into the effects of moderating factors and control variables of age, gender, and past blood donation experiences and their impact on the observed outcomes. Though offering valuable insights into strategies for improving blood donation intentions, our findings may be limited in terms of their generalizability by the high level of education attained by the sample. The educational distribution of adult Greeks might not be fully represented since less than 40% of our sample were undergraduate students while a significant proportion had Master’s and doctoral degrees. This tendency toward higher education levels might be due to the recruitment channels used like academic, professional networks, and social media platforms. As there can exist differences in donation behavior among various educational backgrounds, further investigations are needed on how well these strategies work within a more diversified demographic profile. To ensure that representativeness can be improved upon, future investigations could seek a broader stratification of participants. One of the shortcomings of TPB is that it relies exclusively on individual reports to anticipate behavioral patterns. According to Armitage and Conner [21,47], these responses may contain stereotyped biases or fail to accurately convey people’s true intentions or perspectives. When implementing TPB, the primary focus is on conscious aspects of behavioral dispositions rather than cognitive processes, which can have a greater effect on behavior but be limited in their measurement and evaluation. Time is highlighted as an essential aspect which could confound the relationship between intentions and actual behavior. Future research could focus on examining the changes over time in individuals’ intentions and perceived control regarding blood donation, to gain a greater overview of how these interactions influence the actual manifestation of the blood donation behavior over specific periods of time [22]. Additionally, integrating variables from well-known models of altruistic dispositions, prosocial behavior, or psychological factors could potentially alter the current model and provide interesting directions for future research. As our research approach was based on an online survey with advertisements serving different purposes, from eliciting positive or negative emotions to conveying diverse textual messages, it would be intriguing to investigate how different scenarios impact donor behavior. Diverse blood donation policies and regulations have a considerable influence on donors’ attitudes and behaviors. The effectiveness of distinct motivating incentives, recruiting strategies, and public health initiatives could be evaluated via different communication methods, including experimental techniques, providing diverse results in regard to blood donation intentions.

## Figures and Tables

**Table 1:** Detailed summary of the advertisements.

Emotional Arousal	Textual Message	Number of Ads	Selected Emotions
Positive	Altruistic	3	Joy, Interest, Inspiration
Positive	Egocentric	3	Joy, Interest, Inspiration
Negative	Altruistic	3	Disgust, Guilt, Fear
Negative	Egocentric	3	Disgust, Guilt, Fear
Total Number of ads: 12			

**Table 2:** Sample profile.

		Frequency	Percentage
**Gender**	Male	216	52.2%
	Female	198	47.8%
**Age**	18–25	124	30.0%
	26–30	107	25.8%
	31–40	88	21.3%
	41–50	64	15.5%
	51–60	24	5.8%
	60+	7	1.7%
**Education**	High School Graduate	23	5.6%
	Undergraduate Student	140	33.8%
	Graduate	116	28.0%
	Postgraduate	71	17.1%
	Postgraduate Student	48	11.6%
	PhD Candidate	12	2.9%
	Doctoral Degree Holder	4	1.0%

**Table 3:** Factor loadings, reliability, and convergent validity.

Construct	Items	Factor Loadings	Cronbach’s Alpha	rho_A	CR	AVE
Attitudes towards the advertisement	AAD1	0.774	0.814	0.815	0.878	0.642
	AAD2	0.832				
	AAD3	0.813				
	AAD4	0.785				
Attitudes	ATT1	0.866	0.898	0.917	0.936	0.830
	ATT2	0.925				
	ATT3	0.940				
Blood Donation Anxiety	BDA1	0.928	0.856	0.867	0.913	0.778
	BDA2	0.903				
	BDA3	0.811				
DESNegative	DESNEG1	0.907	0.888	0.891	0.930	0.816
	DESNEG2	0.907				
	DESNEG3	0.896				
DESPositive	DESPOS1	0.765	0.593	0.634	0.779	0.545
	DESPOS2	0.825				
	DESPOS3	0.606				
Behavioral Intention	BI1	0.786	0.799	0.807	0.882	0.715
	BI2	0.904				
	BI3	0.843				

**Table 4:** HTMT ratio.

	AAD	ATT	BDA	BI	DESNEG	DESPOS
AAD						
ATT	0.099					
BDA	0.699	0.072				
BI	0.726	0.167	0.600			
DESNEG	0.463	0.125	0.477	0.583		
DESPOS	0.743	0.105	0.647	0.635	0.472	

**Table 5:** Fornell and Larcker criterion.

	AAD	ATT	BDA	BI	DESNEG	DESPOS
AAD	**0.801**					
ATT	0.084	**0.911**				
BDA	0.587	0.062	**0.882**			
BI	0.590	0.141	0.501	**0.846**		
DESNEG	0.395	0.112	0.420	0.496	**0.903**	
DESPOS	0.550	0.032	0.476	0.452	0.329	**0.738**

**Table 6:** Hypothesis testing.

Hypothesis	Path	Coefficient (β)	SD	*t*-Value	*p*-Value	Results
H1	ATT -> BI	0.084	0.039	2.173	0.030	Supported
H2	BDA -> BI	0.261	0.053	4.959	0.000	Partially Supported
H3a	BDA -> DESNEG	0.287	0.061	4.705	0.000	Supported
H3b	BDA -> DESPOS	0.234	0.051	4.586	0.000	Supported
H4a	DESPOS -> BI	0.226	0.047	4.766	0.000	Supported
H4b	DESNEG -> BI	0.303	0.046	6.515	0.000	Partially Supported
H5a	AAD -> DESNEG	0.227	0.056	4.060	0.000	Supported
H5b	AAD -> DESPOS	0.413	0.050	8.308	0.000	Supported

**Table 7:** Mediation analysis.

Hypotheses	Direct Effects	Coefficient (β)	*t*-Value	*p*-Value	Results
	BDA -> BI	0.261	4.959	0.000	
	ADD -> BI	0.344	7.163	0.000	
	**Total Effects**	Coefficient (β)	*t*-value	*p*-value	
	AAD -> BI	0.162	5.234	0.000	
	BDA -> BI	0.401	8.878	0.000	
	**Specific Indirect Effects**	Coefficient (β)	*t*-value	*p*-value	
H6a	AAD -> DESPOS -> BI	0.093	3.630	0.000	Supported
H6b	AAD -> DESNEG -> BI	0.069	3.094	0.002	Partially Supported
H7a	BDA -> DESPOS -> BI	0.053	3.496	0.000	Supported
H7b	BDA -> DESNEG -> BI	0.087	4.093	0.000	Partially Supported

## Data Availability

The raw data collected by the survey are available upon request to the corresponding author.

## Appendix A. Measurement Items Used for Data Collection

**Attitudes towards the Advertisement (Five-Point Semantic Differential Scale) (AAD)**		
AAD1	Like the ad (1 = I dislike the ad—5 = I like the ad)	Originally Holbrook and Batra Adapted from Ranganathan and Henley
AAD2	Favorable (1 = I react unfavorably to the ad—5 = I react favorably to the ad)	
AAD3	Positive (1 = I feel negative toward the ad—5 = I feel positive toward the ad)	
AAD4	Advertisement is good (1 = The ad is bad—5 = The ad is good)	
AAD5	Information (1 = The ad is uninformative—5 = informative)	
**Emotional Arousal (DES)**		
*Positive Emotions (DESPositive)*		
DESPOS1	I felt glad, happy, joyful.	Galanakis,
DESPOS2	I felt interested, alert, curious.	
DESPOS3	I felt inspired, uplifted, elevated.	
*Negative Emotions (DESNegative)*		
DESNEG1	I felt scared, fearful, afraid.	Galanakis,
DESNEG2	I felt disgust, distaste, revulsion.	
DESNEG3	I felt contemptuous, scornful, disdainful.	
**Blood Donation Anxiety (BDA)**		
BDA1	In the future if I donate blood, I would feel nervous	Robinson 2009 and Chen 2022
BDA2	In the future if I donate blood, I would feel distressed	
BDA3	In the future if I donate blood, I would feel anxious	
**Attitudes (ATT)**		
ATT1	For me, voluntary blood donation, (1 = unsatisfying—5 = satisfying)	
ATT2	For me, voluntary blood donation, (1 = pointless—5 = worthwhile)	
ATT3	For me, voluntary blood donation, (1 = unrewarding—5 = rewarding)	
ATT4	For me, voluntary blood donation, (1 = stressful—5 = relaxing)	
**Behavioral Intention to Donate Blood (BI)**		
BI1	I intend to donate blood in the next 3 months	
BI2	I will donate blood in the next 3 months	
BI3	I would like to donate in the next 3 months	
